# The Mannose Receptor Is Involved in the Phagocytosis of Mycobacteria-Induced Apoptotic Cells

**DOI:** 10.1155/2016/3845247

**Published:** 2016-06-20

**Authors:** Teresa Garcia-Aguilar, Patricia Espinosa-Cueto, Alejandro Magallanes-Puebla, Raúl Mancilla

**Affiliations:** Departamento de Inmunología, Instituto de Investigaciones Biomédicas, Universidad Nacional Autónoma de México, Ciudad Universitaria, 04510 México City, DF, Mexico

## Abstract

Upon* Mycobacterium tuberculosis *infection, macrophages may undergo apoptosis, which has been considered an innate immune response. The pathways underlying the removal of dead cells in homeostatic apoptosis have been extensively studied, but little is known regarding how cells that undergo apoptotic death during mycobacterial infection are removed. This study shows that macrophages induced to undergo apoptosis with mycobacteria cell wall proteins are engulfed by J-774A.1 monocytic cells through the mannose receptor. This demonstration was achieved through assays in which phagocytosis was inhibited with a blocking anti-mannose receptor antibody and with mannose receptor competitor sugars. Moreover, elimination of the mannose receptor by a specific siRNA significantly diminished the expression of the mannose receptor and the phagocytosis of apoptotic cells. As shown by immunofluorescence, engulfed apoptotic bodies are initially located in Rab5-positive phagosomes, which mature to express the phagolysosome marker LAMP1. The phagocytosis of dead cells triggered an anti-inflammatory response with the production of TGF-*β* and IL-10 but not of the proinflammatory cytokines IL-12 and TNF-*α*. This study documents the previously unreported participation of the mannose receptor in the removal of apoptotic cells in the setting of tuberculosis (TB) infection. The results challenge the idea that apoptotic cell phagocytosis in TB has an immunogenic effect.

## 1. Introduction

Macrophages (MØ) and nonprofessional cells are in charge of eliminating apoptotic cells. In the first step, dead cells may release soluble factors that attract MØ, including lysophosphatidylcholine [1], the chemokine MCP-1 [2], ATP, and UTP [3]. The release of membrane vesicles that carry chemotactic factors has also been reported [4–6]. When apoptotic cells and macrophages come in contact, phagocytosis proceeds through the interaction between macrophage receptors and ligands exposed on the surface of target cells. Due to severe derangement of the cell architecture during apoptosis, molecules that are normally inside the cell become exposed on the cell surface. The best studied receptor is phosphatidylserine, which is translocated to the external leaflet of the plasma membrane [7], where it may bind specific macrophage receptors, such as the brain-specific angiogenesis inhibitor 1, stabilin 2, and members of the T cell immunoglobulin mucin domain protein family, which include TIM1, TIM3, and TIM4 [8–11]. Also, opsonin-like molecules with affinity for phosphatidylserine have been identified, and these include milk fat globule-EGF factor 8 [12], protein S [13], and GAS6 [14]. In turn, these molecules are recognized by specific receptors in phagocytic cells. Phosphatidylserine-independent phagocytosis also occurs, and calreticulin translocated from the endoplasmic reticulum to the cell surface during apoptosis can promote phagocytosis via CD91 [15]. C1q may bind apoptotic cells via its globular head domain, which results in complement activation and phagocytosis through C4b and C3b receptors [16]. The redundancy of pathways to eliminate dead cells reflects its importance in cell homeostasis. Deficient removal may result in chronic inflammation and autoimmune disease [17, 18]. In comparison with the great interest aroused by phosphatidylserine and other molecules, few studies have aimed to assess the role of exposed carbohydrates and lectin-like receptors in apoptotic cell phagocytosis. Recently, there has been great interest in the study of host cell apoptosis in* Mycobacterium tuberculosis* (Mtb) infection, partly due to its possible role in innate immunity; however, little is known regarding the mechanisms involved in apoptotic cell removal [19]. In this study, we performed binding inhibition assays and siRNA methodology to determine the role of the mannose receptor (MR) in the phagocytosis of apoptotic MØ induced with mycobacterial cell walls, which contain LpqH, the apoptogenic Mtb glycolipoprotein.

## 2. Materials and Methods

### 2.1. Culture of Transformed* Mycobacterium smegmatis* Strain to Obtain Mycobacterial Cell Walls

The* Mycobacterium smegmatis* strain (mc2155) transformed by electroporation to express LpqH, the 19-kDa Mtb glycolipoprotein (Msmeg/LpqH), was kindly donated by Y. Zhang (MRC Tuberculosis and Related Infections Unit, Hammersmith Hospital, London, UK). The mycobacteria were grown for 5–7 days in Middlebrook 7H9 medium supplemented with 2% glucose and hygromycin B (50 *µ*g/mL). Because LpqH is a cell wall-located glycolipoprotein with apoptogenic properties [20, 21], to induce apoptosis, mycobacterial cell walls were obtained by sonication of bacilli at 60 KHz in iced water (1 min 20 cycles). Thereafter, the sonicate was centrifuged at 15000 to separate the cell wall fraction in the precipitate from the cytosolic protein in the supernatant. The protein content was estimated by the Lowry method. To determine the expression of LpqH, 5 *µ*g of cell wall proteins was separated by 15% SDS-PAGE and transferred to nitrocellulose membranes, and the presence of LpqH was verified by immunoblotting with a mAb (kindly donated by Colorado State University).

### 2.2. Induction of Apoptosis in Bone Marrow-Derived Macrophages and Demonstration of Mycobacterial Proteins in Apoptotic Cells

Bone marrow-derived MØs were obtained from the tibiae and femurs of Balb/c-J AN mice, and the cells were cultured in RPMI-1640 supplemented with 10% heat-inactivated fetal calf serum, 1% essential amino acids, 1% sodium pyruvic acid, 1 mM L-glutamine, and 1% antimycotic (Gibco BRL Products, Rockville, MD, USA). The cells were cultured at 37°C with 5% CO_2_ for ten days in Petri dishes (Costar, Corning Incorporated, NY, USA). The cells were grown to confluence and removed with a scraper, and their viability was assessed by Trypan blue exclusion. To induce apoptosis, 5 × 10^5^ cells were incubated for 24 h with 50 *µ*g of cell wall proteins obtained by sonication from Msmeg-LpqH bacilli. Also, apoptosis was induced exposing the cells to ultraviolet light (UV) for 1 h. To verify apoptosis, the cells were incubated with Annexin V labeled with FITC at room temperature for 15 min. To verify necrosis cells were stained with propidium iodide following manufacturer's instructions. For microscopy, cytospin slides were prepared, mounted with ProLong Gold Antifade with DAPI (Invitrogen, Eugene, OR, USA), and examined with an Olympus BX51 epifluorescence microscope. For flow cytometry, the cells were rinsed extensively with binding buffer and analyzed with a Beckton Dickinson cytofluorometer (San Diego, CA, USA). The apoptotic cell protein content was estimated by the Lowry method. To investigate the presence of mycobacterial antigens in apoptotic MØs, whole cell proteins were separated by 15% SDS-PAGE, transferred to PVDF membranes, and incubated for 3 h with a rabbit antiserum to* M. smegmatis* cell walls diluted 1 : 200 overnight at 4°C or with a mAb to LpqH diluted 1 : 200. After rinsing, the membranes were incubated with horseradish peroxidase-conjugated secondary antibodies for 1 h. A similar immunoblot procedure was followed to characterize the protein profile of the Msmeg-LpqH cell walls. The reactive bands were visualized by chemiluminescence with a SuperSignal West Dura kit (Pierce Biotechnology).

### 2.3. Phagocytosis Assays of Apoptotic Cells and Analysis by Immunofluorescence Microscopy and Flow Cytometry

The Balb/c-derived murine macrophage-like tumor cell line J-774A.1 was obtained from the American Type Culture Collection (Manassas, VA, USA) and cultured as described for the Balb/c bone marrow MØs. For phagocytosis assays, apoptotic MØs were first isolated by rinsing for 5 min at 453 g and subsequent incubation with Annexin V-coated magnetic beads, as indicated by the manufacturer (Miltenyi Biotec, Germany), and 90–95% of the isolated cells were positive for Annexin V, as shown by flow cytometry. For phagocytosis assays, the isolated apoptotic MØs were labeled green with PKH-67 (Sigma-Aldrich), and the J-774A.1 phagocytic cells were labeled red with PKH-26. The J-774A.1 cells (0.5 × 10^6^) were plated and incubated with 50 *µ*g of apoptotic MØs at 37°C for 2, 4, 12, and 24 h. After extensive rinsing with PBS, the cytospin slides were examined with an epifluorescence microscope and a Zeiss LSM 5 Pascal laser-scanning confocal microscope equipped with a mercury lamp and fitted with Ar, HeNe 543 nm, and HeNe 633 nm lasers using the LSM5 Pascal 2.8 software. Phagocytosis was analyzed by flow cytometry as described previously in Section 2.

### 2.4. Phagosome Maturation after Phagocytosis of Apoptotic Macrophages

To verify the acidification of phagosomes with engulfed apoptotic MØs, phagocytosis assays were performed with 1 × 10^6^ PKH-67 labeled phagocytic J-774A.1 cells and 50 *μ*g of the target apoptotic MØs labeled with the pH-sensitive stain pHrodo-SE (Invitrogen) for 24 h at 37°C in serum-free culture medium. For microscopic examination, the cytospin slides were prepared as described above and examined by epifluorescence and confocal microscopy. The phagocytosis of pHrodo-labeled apoptotic MØs was examined by flow cytometry as described above. The settings and compensation levels were optimized using unlabeled samples. To further assess the maturation status of phagosomes, endocytic markers were searched after apoptotic cell phagocytosis. The cells were cocultured with apoptotic MØs labeled in green with PKH-67. After 15, 30, and 60 min, the cells were collected, extensively rinsed with PBS, and permeabilized with 0.025% saponin diluted in PBS/1% bovine fetal serum. After rinsing, the cells were incubated with an anti-CD16-CD32 mAb to block Fc-antibody binding (BioLegend, San Diego, CA, USA). After extensive washing, the phagocytic cells were incubated with a mAb to Rab5 or LAMP1 diluted 1 : 100 for 1 h at 4°C. After rinsing with PBS, a secondary anti-IgG Cy5-labeled antibody diluted 1 : 1000 was added to the cells, and the cells were incubated for 30 min in the dark (BioLegend, San Diego, CA, USA). The cells were examined with an epifluorescence microscope and a confocal microscope.

### 2.5. Immunofluorescence Studies to Assess the Expression of the Mannose Receptor by J-774A.1 Macrophage-Like Cells and Its Role in Apoptotic Cell Phagocytosis

Because the expression of the MR by J774A.1 cells has been questioned [22, 23], we studied its expression by flow cytometry and confocal microscopy using an anti-human mAb that cross-reacts with the murine MR [24]. For flow cytometry, 1 × 10^6^ cells were incubated with a mAb to CD16/CD32, rinsed, and then incubated with an anti-MR antibody diluted 1 : 1000 for 1 h (clone 15-2, isotype IgG1; BioLegend, San Diego, CA, USA). After rinsing with PBS, a secondary Alexa 488-labeled antibody diluted 1 : 1000 was added. For confocal microscopy, the cytospin slides were fixed with 1% paraformaldehyde and incubated with the anti-MR antibody diluted 1 : 100 and then with the secondary Cy5-labeled antibody diluted 1 : 1000. For the control slides, the primary antibody was omitted. The slides were covered with ProLong antifade (Invitrogen) and examined with a laser-scanning confocal microscope. Cy5 excitation was at 632–635 nm and emission was at 666 nm. To assess the role of the MR in apoptotic MØ phagocytosis, we conducted confocal microscopy colocalization studies. Target cells were labeled in green with PKH-26, and the phagocytosis assay was performed as described above. After 4 h of phagocytosis, the cells were fixed in 1% paraformaldehyde in PBS for 10 min, permeabilized with 0.1% Triton X-100 for 5 min, and incubated with the mAb to Fc and then for 1 h with the anti-MR antibody diluted 1 : 100. A secondary anti-IgG antibody labeled with Alexa 488 diluted 1 : 1000 was used. Cytospin slides were prepared and examined by epifluorescence and confocal microscopy.

### 2.6. Competitive Inhibition Assays to Determine the Role of the Mannose Receptor in the Phagocytosis of Apoptotic Bodies

Phagocytosis trials were carried out as described previously with the exception that phagocytosis was inhibited by several means. PKH-67-labeled phagocytic J-774A.1 cells (0.5 × 10^6^) were preincubated with 50 *µ*M N-acetylglucosamine (GlcNAc) or 5 mg of mannan (Sigma-Aldrich). After 30 min and without rinsing, 50 *µ*g of PKH-26-labeled apoptotic MØs was added to the culture. In similar experiments, phagocytic cells were preincubated with 5 or 10 *μ*g of anti-MR mAb before apoptotic MØs were added. As a control, a similar incubation was carried out with an isotype control antibody. The effects of these inhibition procedures were analyzed by flow cytometry as described previously.

### 2.7. siRNA Silencing of the Mannose Receptor and Its Effects on the Phagocytosis of Apoptotic Macrophages

To study the effects of MR gene silencing, the cells were transfected with MR siRNA with the sequence GAACAAAGAUCCACUGACU (Thermo Scientific, Rockford, IL, USA). For transfection, J-774A.1 cells (0.2 × 10^6^) were cultured in reduced serum medium OptiMEM (Gibco) and treated with Oligofectamine according to the manufacturer's instructions. The cells were then incubated with 60 pmol of siRNA specific for the MR for 64 h. Following the previously described procedures, the expression of the MR and the phagocytic capacity of siRNA-silenced J-774A.1 cells were assessed by flow cytometry. Similar procedures were followed in control experiments with cells that were transfected with a scramble siRNA (Thermo Scientific).

### 2.8. Macrophage Cytokine Production after Apoptotic Cell Phagocytosis

The cytokine release in the supernatants of J-774A.1 cells which were cocultured with apoptotic MØs for 24 h was analyzed. Following the manufacturer's instructions, a sandwich ELISA was performed with mAb to IL-12, TNF-*α*, TGF-*β*, and IL-10 (BioLegend). To determine if apoptotic macrophages release by themselves cytokines, we incubated apoptotic macrophages isolated with Annexin V coated microbeads for 24 h. The levels of cytokines released in the culture medium were determined by measuring the absorbance at 450 nm with a microplate reader. The cytokine concentrations in the samples were calculated using standard curves generated from recombinant cytokines, and the results are expressed in pg/mL.

### 2.9. Statistics

Normally distributed data are expressed as the means ± SD and assessed for significance by Student's *t*-test. Nonparametric Mann-Whitney and Kruskal-Wallis with Dunn's multiple comparison tests were used with GraphPad Prism software (version 5.01; San Diego, CA, USA). Statistical significance was assumed with *p* values ≤ 0.05.

### 2.10. Ethics Statement

Use of animals and experimental procedures were reviewed and approved by the Bioethics Committee of our Institute following established protocols.

## 3. Results 

### 3.1. Induction of Macrophage Apoptosis with Mycobacterial Cell Walls

**Figure 1:**
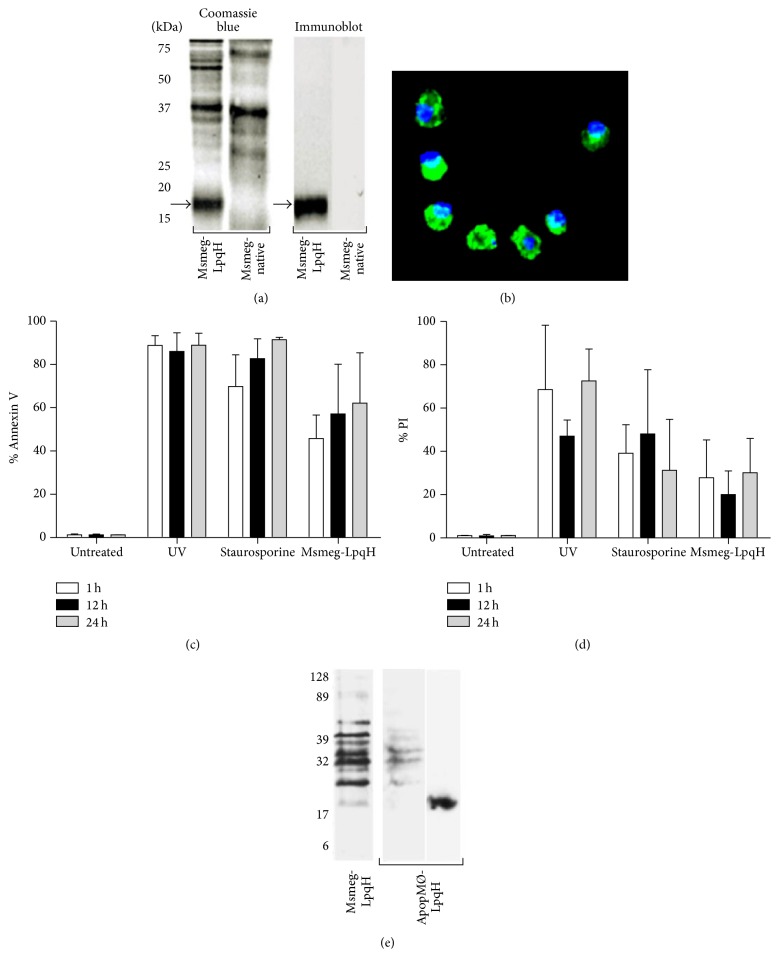
Mycobacterial cell walls mediate the apoptosis of bone marrow macrophages. Demonstration of mycobacterial proteins in apoptotic cells. The cell wall of the transformed* M. smegmatis* strain (Msmeg-LpqH) expresses LpqH, the 19-kDa Mtb glycolipoprotein ((a), arrows). The native strain does not express the protein. Bone marrow MØs treated with mycobacterial cells that carry LpqH develop apoptosis, as verified by epifluorescence ((b), original 40x) and flow cytometry with FITC-labeled Annexin V (c). With staurosporine and UV, higher levels of apoptosis were observed (c). High level necrosis as revealed with propidium iodide was observed (d). Immunoblotting of mycobacteria-induced apoptotic MØs (ApopMØ-LpqH) with an anti*-M. smegmatis* antiserum and with a mAb revealed the presence of a few* M. smegmatis* antigenic bands (left) and LpqH (right). Msmeg-LpqH, protein profile of the* M. smegmatis* cell wall used to induce apoptosis (e). UV, ultraviolet light. We show representative results of three independent experiments.

Bone marrow-derived MØs from Balb/c-J AN mice were treated for 1, 12, and 24 h with cell walls from an* M. smegmatis* strain transformed to express LpqH (Msmeg-LpqH), the Mtb glycolipoprotein [21, 25]. Similar to other mycobacterial lipoproteins [20], LpqH is strongly expressed in the bacterial cell wall, as shown in Coomassie blue-stained gels and by immunoblot with a specific mAb (Figure 1(a), arrows). Cell walls of native* M. smegmatis* do not express LpqH (Figure 1(a)). MØs treated with Msmeg-LpqH cell walls developed high levels of apoptosis, as demonstrated by epifluorescence microscopy of cytospin slides stained with Annexin V/FITC (Figure 1(b)). As determined by flow cytometry with Annexin V, 60% cell apoptosis was observed at 24 h (Figure 1(c)). UV was used as a control method to induce apoptosis without the participation of foreign antigens, and staurosporine was used as a positive control. After UV and staurosporine treatment, the apoptosis levels were higher than those observed with mycobacterial cell walls (Figure 1(c)). Apoptotic MØs were isolated with magnetic beads coated with Annexin V. Propidium iodide staining showed that UV and staurosporine induced high necrosis levels, particularly at 24 h. With Msmeg/LpqH cell wall necrosis was less intense (Figure 1(d)). To determine whether the mycobacterial proteins used to trigger apoptosis were incorporated into apoptotic bodies, immunoblotting performed using an anti-*M. smegmatis* rabbit antiserum revealed that some of the antigenic bands of the Msmeg-LpqH cell wall (Figure 1(e)) were present in apoptotic MØs induced with Msmeg-LpqH cell walls (ApopMØ-LpqH) but not in those induced with UV. LpqH was demonstrated in apoptotic cells with the anti-IT-19 mAb (Figure 1(e)).

### 3.2. Phagocytosis of Apoptotic Cells by J-774A.1 Macrophage-Like Cells

**Figure 2:**
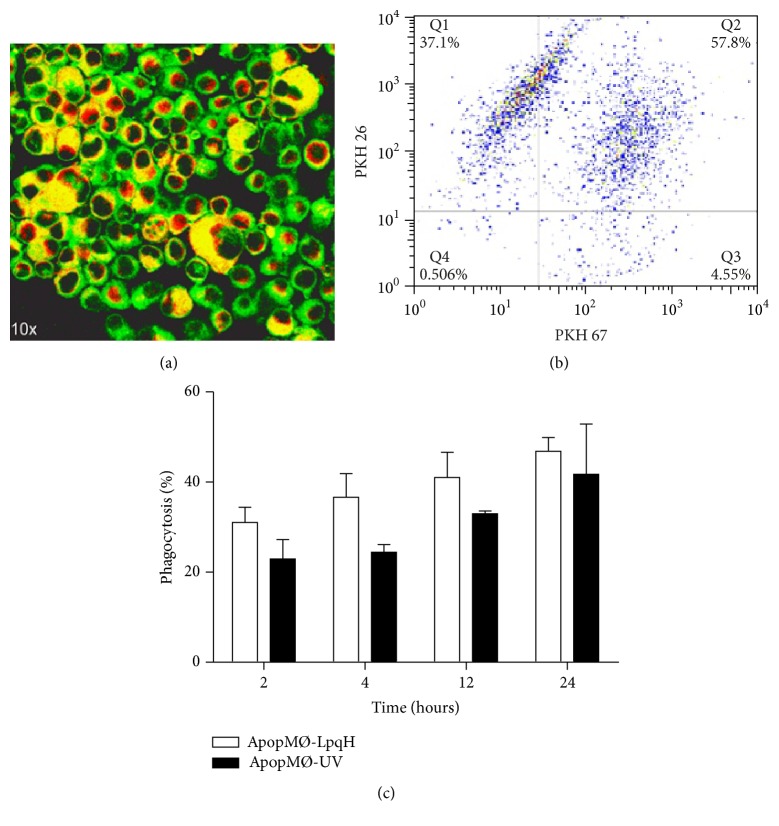
Phagocytosis of apoptotic macrophages assessed by epifluorescence and flow cytometry. We conducted phagocytosis assays with J-774A.1 phagocytic cells labeled with PKH-67 (green fluorescence) and apoptotic MØs labeled with PKH-26 (red fluorescence). After two hours of incubation, in overlaid images, the confocal examination of mid-sectioned phagocytic cells showed enlarged MØs containing abundant yellow-fluorescent apoptotic bodies ((a), original 40x). Engulfed whole cells were not observed, perhaps due to their degradation within phagolysosomes. A representative dot blot shows double-labeled phagocytic cells in the upper right quadrant (b). Phagocytosis was quantitated by flow cytometry (c). Time-dependent phagocytosis was observed; that is, greater levels of apoptosis were observed at 24 h (47.7%). The results of three independent experiments are shown.

**Figure 3:**
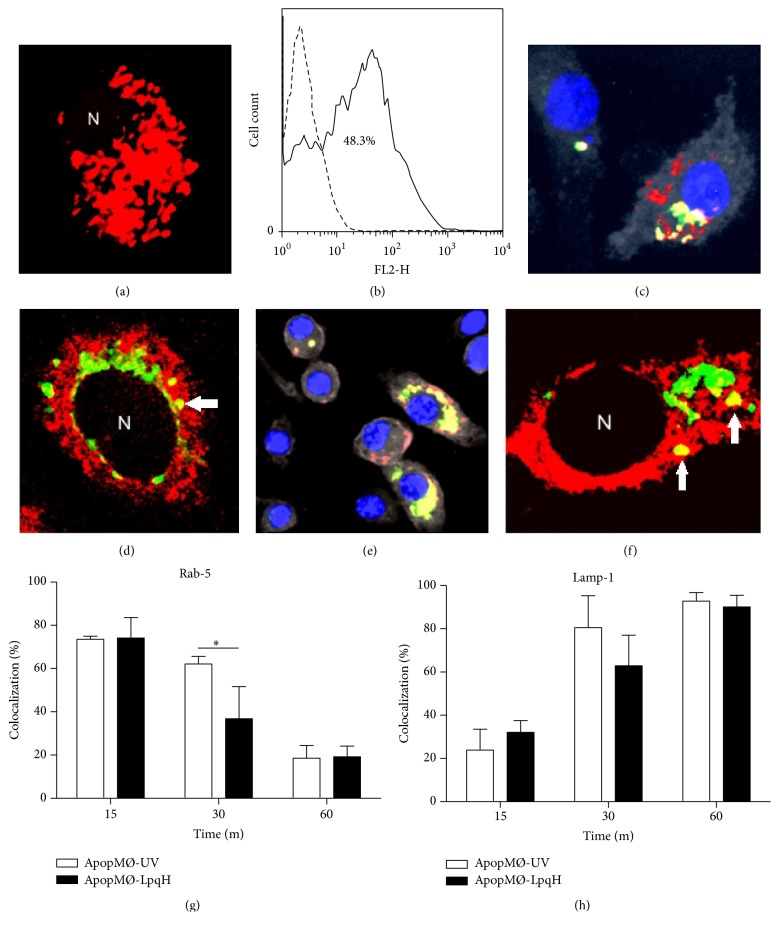
Phagosomes with engulfed apoptotic macrophages mature into phagolysosomes. Phagocytosis assays were conducted with J-774A.1 cells and apoptotic MØs labeled with pHrodo, a marker that emits strong red fluorescence in an acidic environment. After 24 h of phagocytosis, confocal microscopy showed numerous pHrodo fluorescent apoptotic bodies in the majority of the cells ((a), original 100x). As determined by flow cytometry, 48.3% of the cells were found to be positive for pHrodo (b). To further determine phagosome maturation, phagocytic cells were cocultured for 15, 30, and 60 min with apoptotic MØs labeled green with PKH-67. The expression of the early endocytic marker Rab5 and LAMP1, a late endosome marker, was assessed by immunofluorescence. The cells were permeabilized and incubated with Cy5-labeled antibodies to Rab5 or LAMP1. In overlaid images, epifluorescence ((c), original 60x) and confocal microscopy ((d), original 100x) show cells containing yellow-fluorescent vesicles, indicating the colocalization of Rab5 with PKH-67-labeled apoptotic bodies. The colocalization of LAMP1 and engulfed apoptotic MØs was also demonstrated (e and f). As expected, the recruitment processes of Rab5 and LAMP1 followed inverse time courses (g and h). The results of three independent experiments are shown. Student's *t*-test was used to assess the statistical significance. ^*∗*^ *p* < 0.05. N, nucleus.

Bone marrow-derived MØs rendered apoptotic by UV (ApopMØ-UV) or ApopMØ-LpqH were isolated first by 1500 rpm centrifugation and then with Annexin V-coated microbeads. Apoptotic MØs were labeled with PKH-26 (red fluorescence) and cocultured with J-774A.1 phagocytic cells labeled with PKH-67 (green fluorescence). Confocal microscopy of multiple mid-sectioned cells was conducted. After two hours of phagocytosis, in the overlaid images, we observed enlarged cells containing abundant yellow fluorescent material with a nodular appearance consistent with apoptotic bodies (Figure 2(a)). The absence of whole engulfed apoptotic cells suggests their degradation, a possibility supported by our assays showing that phagolysosome fusion occurs as soon as 15 min after phagocytosis of apoptotic cells (see Figures 3(g) and 3(h)). Phagocytosis was assessed by cytofluorometry (Figures 2(b) and 2(c)), and time-dependent phagocytosis was observed because a greater degree of phagocytosis was observed at 24 h (47.7%). The phagocytosis of ApopMØ-UV was similar (Figure 2(c)).

### 3.3. Phagosomes with Ingested Apoptotic Cells Mature to Phagolysosomes

Following phagocytosis, the phagosome matures into phagolysosome through a process that depends on the sequential fusion of endosomes and lysosomes with the phagosomes [26]. The phagolysosome is an acidic vesicle with a pH below 5.5 rich in hydrolytic enzymes. The low pH is due to the action of a vacuolar-type H-ATPase. After acidification, phagosomes undergo fusion with lysosomes [26]. Mtb can block phagolysosomal fusion [27]; hence, we wanted to determine the maturation status of phagosomes containing apoptotic cells induced with mycobacterial cell walls; these apoptotic bodies carry mycobacterial proteins (Figure 1(d)). First, we analyzed the acidity of phagosomes with apoptotic bodies labeled with pHrodo, a pH-sensitive dye that emits a strong red fluorescence in an acidic environment [28]. Membrane-bound particles do not fluoresce, thus improving the accuracy of phagocytosis quantification. Within 24 h of phagocytosis, confocal microscopy showed that most of the cells contained abundant pHrodo-fluorescent apoptotic bodies (Figure 3(a)). As determined by flow cytometry, 48.3% of the cells were pHrodo positive (Figure 3(b)). An autofluorescence control is shown (dashed lines). In phagosome maturation, the participation of small GTPases is critical [26]; therefore, we searched for the expression of Rab5, an early phagosome marker, and LAMP1 which characterizes phagolysosome fusion [29]. Epifluorescence (Figure 3(c)) and confocal microscopy (Figure 3(d), arrow) showed the colocalization of apoptotic bodies with Rab5 in small phagocytic vesicles of most cells (yellow fluorescence, arrows). The colocalization of LAMP1 and engulfed apoptotic bodies was also demonstrated (Figures 3(e) and 3(f)). To quantify the endocytic marker recruitment, multiple 20x epifluorescence photomicrographs were analyzed, and the cells with engulfed apoptotic bodies expressing Rab5 (Figure 3(g)) or LAMP1 (Figure 3(h)) were counted. Within 15 min of phagocytosis, 75.2% of the cells contained Rab5-positive phagosomes; after that, Rab5 acquisition declined to 19.3% at 60 min. The delivery of LAMP1 followed an inverse course, increasing over time from 28.7% at 15 min to 90.8% at 60 min. Phagosomes containing ApopMØ-UV followed a similar maturation process, although the percentage of Rab5-expressing phagosomes at 30 min was significantly less than that observed with mycobacteria-induced apoptotic cells (*p* < 0.05). Altogether, these findings document the maturation of phagosomes that have engulfed apoptotic MØs. The results of three independent experiments are presented.

### 3.4. The Mannose Receptor Participates in the Phagocytosis of Apoptotic Macrophages, as Demonstrated by Immunofluorescence

**Figure 4:**
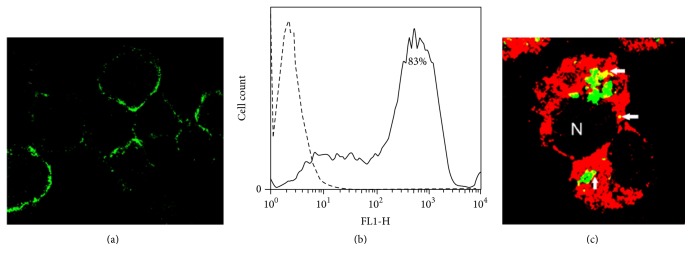
Immunofluorescence demonstration of the role of the mannose receptor in the phagocytosis of apoptotic cells. To determine the expression of the MR on J-774A.1 macrophage-like cells, the cells were incubated with a Cy5-labeled anti-MR antibody. Confocal microscopy revealed patchy membrane fluorescence in many of the cells ((a), original 40x). A representative flow cytometry histogram demonstrated 83% MR expression (b); an isotype control is shown (dashed line). In overlaid confocal microscopy images, the role of the MR in phagocytosis was shown by the yellow fluorescent colocalization (arrows) of the MR (red fluorescence) with apoptotic bodies (green fluorescence) ((c), original 100x).

The expression of the MR by J-774A.1 cells has been questioned [22, 23]. Hence, we conducted immunofluorescence studies by incubating cells with an anti-MR antibody labeled with FITC. Confocal microscopy showed that the majority of the cells exhibited patchy membrane fluorescence (Figure 4(a)). Flow cytometry studies of several cell cultures revealed wide variations in the MR cell surface expression, ranging from 20 to 90%, and a representative histogram is shown (Figure 4(b)). As determined by assays to investigate the role of the MR in apoptotic cell removal, after 4 h of phagocytosis of PKH-67-labeled ApopMØ-LpqH, phagocytic J-774A.1 cells were permeabilized and incubated with an anti-MR mAb and then with a secondary antibody labeled with Cy5 (red fluorescence). As determined by confocal microscopy, 65% of the cells showed cytoplasmic yellow-fluorescent vesicles, indicating the colocalization of the MR with apoptotic MØs (Figure 4(c), arrows). Similar observations were done with ApopMØ-UV (not shown).

### 3.5. Inhibition of the Phagocytosis of Apoptotic MØs in Competitive Inhibition Assays and with an Antibody to the Mannose Receptor

**Figure 5:**
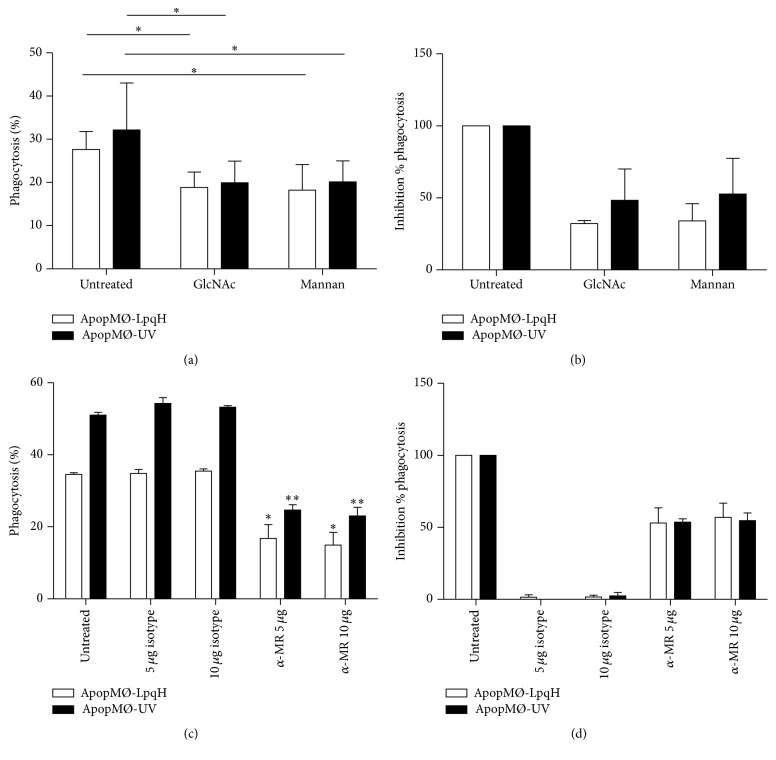
Competitive sugars GlcNAc and mannan and a blocking anti-MR antibody inhibit the phagocytosis of apoptotic cells. As determined by competitive inhibition assays, the preincubation of phagocytic cells with mannan and GlcNAc significantly reduced the degree of phagocytosis, expressed as the percent of cells with engulfed apoptotic bodies (a) or the percent inhibition of the phagocytosis (b). Preincubation with 5 and 10 *µ*g of anti-MR antibody significantly reduced the percent of phagocytic cells (c). The percent inhibition of the phagocytosis is shown. The results of three independent experiments are presented. The isotype control had no inhibitory effects on the phagocytosis. Gaussian Student's *t*-test was used to assess statistical significance (^*∗*^ *p* < 0.05; ^*∗∗*^ *p* < 0.05).

To further characterize the role of the MR, before incubation with apoptotic cells induced with Msmeg-LpqH cell walls and UV, the phagocytic cells were preincubated with mannan and GlcNAc, recognized MR competitors, and a blocking antibody to the MR. Preincubation with 50 mM GlcNAc and with 5 mg mannan reduced significantly the percent phagocytosis of apoptotic cells induced with mycobacteria cell walls and with UV (Figure 5(a), *p* < 0.05). The percent inhibition of the phagocytosis of mycobacteria-induced apoptotic cells with GlcNAc was 35.9% and with mannan it was 27.2% (Figure 5(b)). With 5 and 10 *µ*g of anti-MR antibody, the percent phagocytosis of both mycobacteria cell walls and UV-induced apoptotic cells was reduced significantly (*p* < 0.05, Figure 5(c)). The percent inhibition of the phagocytosis of Msmeg/LpqH-induced apoptotic cells by macrophages preincubated with 5 *µ*g anti-MR antibody was 53% and with 10 *µ*g it was 56.9% (Figure 5(d)).

### 3.6. Inhibition of the Expression of the MR by Small Interfering RNA and Its Effect in the Phagocytosis of Apoptotic Macrophages

**Figure 6:**
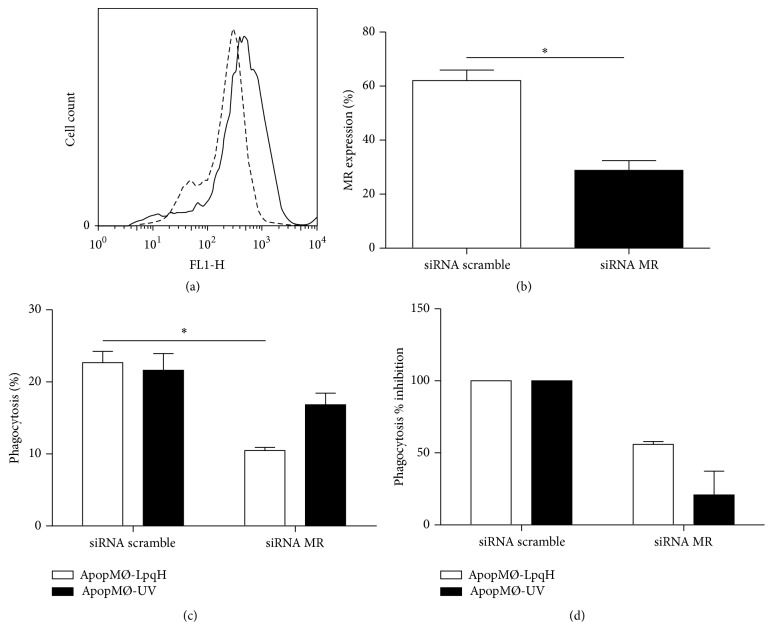
Mannose receptor specific small interference RNA decreases mannose receptor expression and the phagocytosis of apoptotic cells. J-774A.1 cells were transfected with siRNA specific for the MR for 64 h. The expression of the MR and the phagocytic ability of cells transfected with siRNA MR and with a scrambled control are shown. The cell surface expression of the MR was assessed by flow cytometry. In a representative histogram (a) the inhibition induced is shown (dashed lines). In comparison with the MR expression of cells treated with scramble siRNA, MR-specific siRNA reduced significantly MR expression (b). Compared with cells treated with scrambled sRNA, phagocytosis of apoptotic cells was significantly reduced in MØs transfected with specific siRNA expressed as the percent of phagocytic cells (c) or the percent inhibition of the phagocytosis (d). The results of three independent experiments are presented. The statistical significance was assessed by one-way ANOVA, unpaired *t*-test, Student's *t*-test, and nonparametric Kruskal-Wallis test; ^*∗*^ *p* < 0.05.

To most specifically demonstrate the role of the MR in the phagocytosis of apoptotic cells, a siRNA approach was undertaken. Compared with untreated cells or cells receiving scramble siRNA, the treatment of J-774A.1 cells with MR-specific siRNA resulted in a significant decrease in the expression of the MR (Figures 6(a) and 6(b), *p* < 0.05). Moreover, a significant reduction in the rate of phagocytosis of apoptotic walls induced with Msmeg-LpqH was also induced (Figure 6(c), *p* < 0.05).This corresponds to 53.6% inhibition (Figure 6(d)). Decreased phagocytosis of apoptotic cells induced by UV was also observed with less significance. These findings strongly indicate the role of the MR in the phagocytosis of apoptotic cells induced with mycobacterial cell walls or UV.

### 3.7. Apoptotic Cells Upregulate the Secretion of the Anti-Inflammatory Cytokines TGF-*β* and IL-10. 

**Figure 7:**
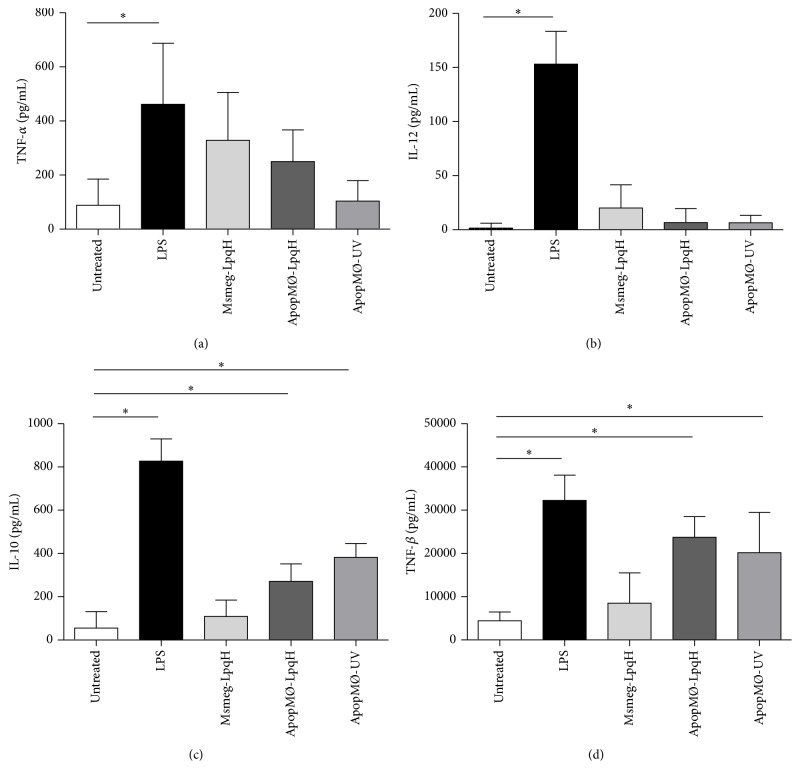
The phagocytosis of apoptotic MØs upregulates anti-inflammatory cytokines in macrophages. A total of 1 × 10^6^ cells/mL were cocultured with 50 *μ*g of apoptotic MØs for 24 h. The culture supernatants were collected and assayed for cytokines using ELISA. The data shown are from four independent experiments. Msmeg-LpqH, cells walls of transformed* M. smegmatis* that expresses LpqH. The statistical significance was determined by one-way ANOVA and Kruskal-Wallis test; ^*∗*^ *p* < 0.05.

It was previously shown that the phagocytosis of apoptotic cells generates an anti-inflammatory cytokine program [30, 31]. Autocrine cytokine production is important due to its role in the polarization of the T cell responses toward a Th1 or Th2 pattern [32]. In this study, we used a capture ELISA method to quantify the cytokines in the culture supernatants of cells that had been cocultured with apoptotic MØs induced with mycobacterial cell walls or UV for 24 h. We used mAbs against the prototypic proinflammatory cytokines TNF-*α* and IL-12, which play a central role in antimycobacterial immunity [32]. We also quantified IL-10 and TGF-*β*, which are known to exert downregulatory effects on T cell-mediated immunity. A modest increase was observed in TNF-*α* production (Figure 7(a)), and IL-12 production was similar to that obtained with the untreated cells (Figure 7(b)). The analysis of anti-inflammatory cytokines showed that IL-10 production was significantly increased in the cells treated with apoptotic bodies (Figure 7(c)). TGF-*β* release was even higher in cells treated with apoptotic cells induced with either mycobacterial cell walls or UV (Figure 7(d)). Apoptotic macrophages isolated with Annexin V did not release cytokines (data not shown).

## 4. Discussion 

Apoptosis occurs extensively during embryonal development and the extrauterine life to maintain stable cell populations [33]. Recently, much attention has been given to its role in cancer, autoimmunity, and infections, such as TB. The past decade has seen a great expansion in our knowledge regarding the pathways of apoptotic cell clearance, and multiple ligand/receptors pairs have been described [17]. In this study, we demonstrate the role of the MR in the phagocytosis of MØ that underwent apoptosis death induced by mycobacterial cell walls carrying LpqH, the apoptogenic Mtb glycolipoprotein [21, 25]. The preincubation of phagocytic cells with mannan and GlcNAc, which are well-known MR competitor sugars, and with a blocking antibody to the MR significantly diminished phagocytosis. Moreover, silencing the MR gene with a small interfering RNA confirmed the role of the MR in the phagocytosis of apoptotic cells. However, phagocytosis inhibition was incomplete suggesting the participation of other ligand/receptor pairs in the removal of dead cells. To the best of our knowledge, this study provides the first description of the specific involvement of the MR in the elimination of apoptotic cells.

Our current observations and the vast literature on the subject show that the removal of dead cells is a highly redundant process in which multiple receptor/ligand systems participate. However, despite the well-known exposure of glycans on the surface of apoptotic cells, little attention has been given to the role played by lectin-like receptors. A pioneering study by Duvall et al. showed that the binding of apoptotic cells to macrophages is inhibited by N-acetylglucosamine, N-acetyl galactosamine, and D-galactose [34]. Later studies described changes in cell surface glycosylation that generate ligands for phagocytosis via lectin-like receptors. The abnormal exposure of mannose, fucose, and GlcNAc was demonstrated using vegetal lectins [35]. These and other studies have suggested the role of surface-exposed sugars in the phagocytosis of apoptotic cells. Blebs that carry immature desialylated endoplasmic reticulum glycoproteins and mannose residues are ingested by macrophages [36]. The preincubation of cells with sugar cocktails, which include fucose, galactose, N-acetylglucosamine, and mannose, inhibits phagocytosis. In these studies, however, the identification of the receptors involved was not achieved [34, 37, 38].

Our current observations may be relevant to the pathogenesis of tuberculosis (TB). In the last decade, much attention has been given to observations showing that macrophages develop apoptosis in response to Mtb infection. In human TB, apoptotic macrophages are observed in granulomas and bronchoalveolar lavages [39, 40]. The biological significance of apoptosis in TB is still far from being completely understood. Growing evidence suggests that it is a macrophage innate immune response to intracellular infection [41]. In TB, the role of unremoved apoptotic cells in inflammation and tissue damage is unknown. In the lung, inflammatory diseases, such as chronic obstructive pulmonary disease, cystic fibrosis, and asthma, large numbers of apoptotic cells are observed in sputum and lung tissue [42]. Increasing evidence suggests that the deficient removal of these cells could contribute to the progression of inflammation in these diseases [42–44].

Phagocytosis allows the internalization of particles, including pathogens and apoptotic cells, by professional and nonprofessional phagocytic cells. Interiorized particles are contained in specialized vesicular structures termed phagosomes. After the closure of the phagocytic cup, phagosomes undergo a maturation process that culminates in the acquisition of the capacity to degrade the ingested cargo [26]. Phagosomes acquire first Rab5 and subsequently Rab7, a marker of late endosomes. In a final step, phagosomes fuse with lysosomes, which are acidic membrane-bound organelles rich in hydrolytic enzymes. The lysosomal membrane possesses a variety of proteins involved in the acidification of the lysosomal lumen and the fusion with endosomes and phagosomes [29]. The most abundant markers are LAMP1 and LAMP2, both of which are involved in the maintenance of the lysosomal membrane integrity and phagolysosome formation [45].

In Mtb infection, bacilli are contained in permissive phagosomes, where they can survive and replicate, due to the ability of mycobacteria to arrest phagosome/lysosome fusion [27]. In this study, we found that phagosomes containing apoptotic MØs that carry mycobacterial antigens mature to phagolysosomes through the sequential acquisition of endocytic markers. Early in this process, the delivery of the early endocytic marker Rab5 to phagosomes was observed in the majority of cells that had engulfed apoptotic MØs and, later in the process, Rab5 acquisition declined markedly. In contrast, the acquisition of LAMP1, a phagolysosome marker, followed an inverse course, with LAMP being present in most of the cells late in the process. These findings are in agreement with those obtained in studies on phagosome maturation in general [26].

There is scant information regarding the events that occur after the phagocytosis of apoptotic cells. Prior to engulfment, Rho GTPases participate in the regulation of actin dynamics; Rac1 is recruited to form phagocytic cups, which consist of actin patches, whereas after apoptotic cell internalization Rac1 is rapidly downregulated [46]. It has been shown that phagosomes containing apoptotic cells acidify faster than phagosomes carrying opsonized viable cells and that a Rho GTPase is involved in this process [47]. Using fluorescence resonance energy transfer, Kitano et al. observed that, during the phagocytosis of apoptotic thymocytes, activated Rab5 is recruited to the nascent phagosome membrane within 3–5 min after disassembly of the actin coat and closure of the phagocytic cup [48]. In this study, Gapex-5 was found to be necessary for Rab5 activation. It has also been shown that Vps34 activates Rab5 by mediating the interaction between Rab5 and dynamin 2 [49]. In the disposal of apoptotic cells, lysosomes play a critical role, and the acidification of phagosomes has been implicated in the degradation of apoptotic cells [50, 51]. Erwig et al. reported the expression of LAMP1 and the late endosomal marker Rab7 in phagosomes containing apoptotic cells but not in phagosomes containing opsonized cells [47]. In another study, the degradation of apoptotic cells was shown to occur within 10 to 20 min [48]. In our study, phagocytosis assays were carried out with whole apoptotic cells, although we observed by confocal microscopy only fragmented apoptotic material consistent with apoptotic bodies. These observations suggest apoptotic cell degradation in keeping with the fact that most phagocytic vacuoles were LAMP1 positive, thus indicating phagolysosome fusion.

Contrary to the proinflammatory response that follows the phagocytosis of pathogens, several studies have shown that the uptake of apoptotic cells mediates an anti-inflammatory response. After the phagocytosis of apoptotic cells, macrophages produce the anti-inflammatory cytokine IL-10 and less of the proinflammatory cytokines TNF-*α*, IL-1b, and IL-12. [30]. In another study, macrophages that had ingested apoptotic cells exhibited decreased production of GM-CSF, IL-1b, IL-8, IL-10, and eicosanoids and increased production of TGF-b1, PGE2, and PAF [31]. It was recently shown that apoptotic cells may also be immunostimulatory [52–54]. In dying cells, molecules with immunogenic properties, including heat shock proteins, tumor antigens, cell surface-exposed calreticulin, and HM0GB1, have been identified [54].

The MR is expressed abundantly on MØs, a cell that plays a central role in the innate response to invading microbes [55]. This receptor recognizes mannose, fucose, and GlcNAc, which may be present on the surface of many pathogens, including Mtb [56]. The phagocytic capacity of the MR has been questioned previously [55], although some studies support such a role. Nonphagocytic COS-1 cells transfected with the MR acquire the ability to phagocytose yeasts and* Pneumocystis carinii* [57, 58]. The uptake of* Francisella tularensis* by J-774A.1 monocytes was documented through inhibition assays with mannan and a blocking antibody to MR [59]. Furthermore, the phagocytosis of unopsonized* Pneumocystis* through the MR has been documented by targeted MR siRNA gene silencing [60]. It has also been reported that the phagocytosis of bacilli by THP-1 cells is inhibited by MR competitor carbohydrates, by calcium depletion, and with a mAb to MR [61]. Regarding the mechanisms of MR phagocytosis, focal F-actin polymerization and small Rho GTPase activation are required [60].

Various observations show that the phagocytosis of pathogens through the MR promotes a permissive intracellular environment by blocking phagolysosome fusion [62] and by the upregulation of anti-inflammatory cytokines and the inhibition of proinflammatory IL-12 and ROS [62–65]. Therefore, the mannose receptor can be considered a highly convenient route of phagocytosis for the maintenance of the removal of cells undergoing apoptotic death as an immunologically silent process.

## 5. Conclusions

This study documents the participation of the macrophage mannose receptor in the phagocytosis of macrophages rendered apoptotic with mycobacterial cell walls that carry LpqH, the apoptogenic* M. tuberculosis* lipoprotein. The study also shows that the phagocytosis of dead cells triggers the production of anti-inflammatory cytokines, thus challenging the idea that apoptotic cell phagocytosis in TB has an immunogenic effect. Here we show that blocking the mannose receptor leads to a decrease in phagocytosis of both Msmeg-LpqH-induced and UV-induced apoptotic macrophages. Therefore, the role of the mannose receptor in the phagocytosis of apoptotic cells seems to be a general biological process and might not be of importance in TB pathogenesis.
